# Rapidly Progressing Osteomyelitis of the Mandible

**DOI:** 10.1155/2013/249615

**Published:** 2013-11-18

**Authors:** Yukiko Kusuyama, Ken Matsumoto, Shino Okada, Ken Wakabayashi, Noritami Takeuchi, Yoshiaki Yura

**Affiliations:** ^1^Department of Dentistry and Oral Surgery, Matsubara Tokushukai Hospital, 7-13-26 Amamihigashi, Matsubara-shi, Osaka 580-0032, Japan; ^2^Department of Oral and Maxillofacial Surgery II, Osaka University, Graduate School of Dentistry, Osaka 565-0871, Japan

## Abstract

Acute osteomyelitis exists as a refractory disease even now, which usually exhibits systemic symptoms such as fever or malaise and local redness or swelling. The present paper describes a case of acute osteomyelitis of the mandible that was rapidly progressing without typical symptoms. The patient had liver cirrhosis, which should be one of the systemic factors that affect immune surveillance and metabolism. Actinomycotic druses and filaments were detected from the sequestrum. These were considered to play a role in the rapid progression of osteomyelitis without typical symptoms. There has been no evidence of local recurrence 24 months after surgery.

## 1. Introduction

Acute osteomyelitis of the jaws is not commonly seen in modern oral and maxillofacial surgery practice. Generally speaking, this can be related to our society having become more health conscious, resulting in an increased awareness of nutrition, as well as earlier and better access to health care than in the past [1, 2]. However, acute osteomyelitis exists as a refractory disease even now, which usually exhibits systemic symptoms such as fever, malaise or high levels of CRP and local redness, swelling, or pus discharge. It is known that osteomyelitis can be attributed to one or more of the predisposing systemic diseases [3]. In immune-compromised patients, it is easy to expect that acute inflammatory reactions are poor. Few case reports such as osteomyelitis of the jaws with poor acute inflammatory reactions and rapid progression have been documented. The present paper describes a case of acute osteomyelitis of the mandible, with liver cirrhosis, that was rapidly progressing without typical symptoms.

## 2. Case Report

A 77-year-old man was referred to our hospital for postextraction hemorrhage and spontaneous pain in the socket of the left mandibular first molar. The patient had a 1-month history of spontaneous pain of the left mandibular first molar. At a nearby dental clinic, restorative treatment was performed. However, as the pain continued, the tooth was finally extracted on January 19, 2011. Next day he visited our hospital.

**Figure 1:**
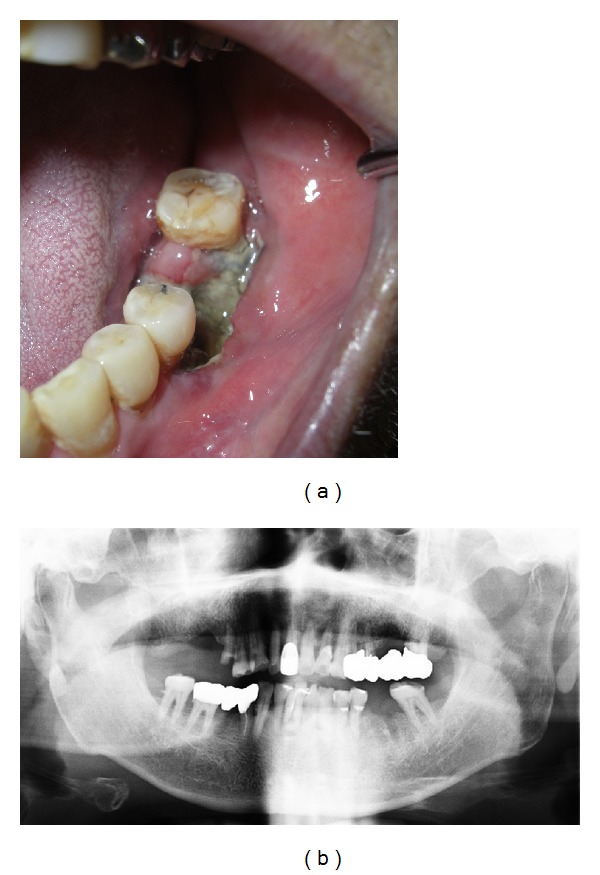
Clinical findings at the initial visit. (a) Close-up view of the socket in the left mandibular first molar region. (b) Panoramic radiograph showing neither abnormal consolidation nor ill-defined trabecular bone structure around the socket and clear running of the inferior alveolar arteries.

**Figure 2:**
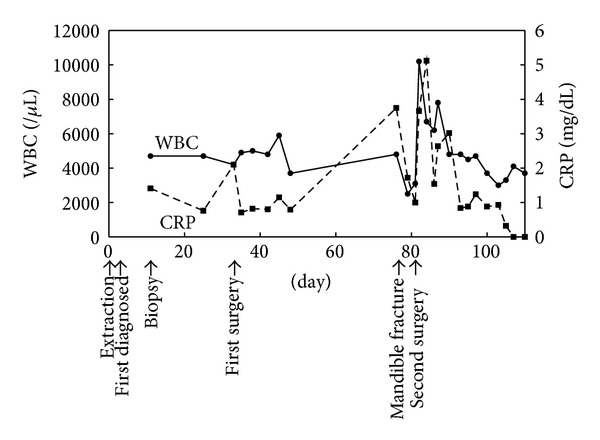
Overview of the clinical events and laboratory data.

**Figure 3:**
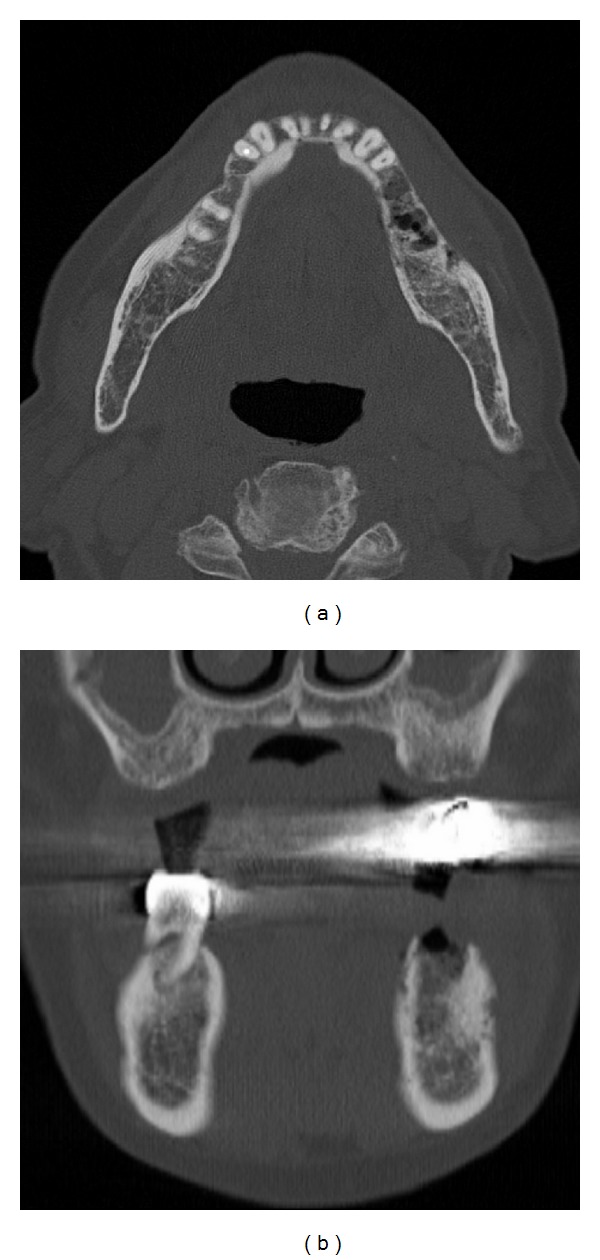
CT scans at 14 days after the initial visit showing remarkable absorption of the cortical bone in the left mandibular molar region. (a) Axial section. (b) Coronal section.

**Figure 4:**
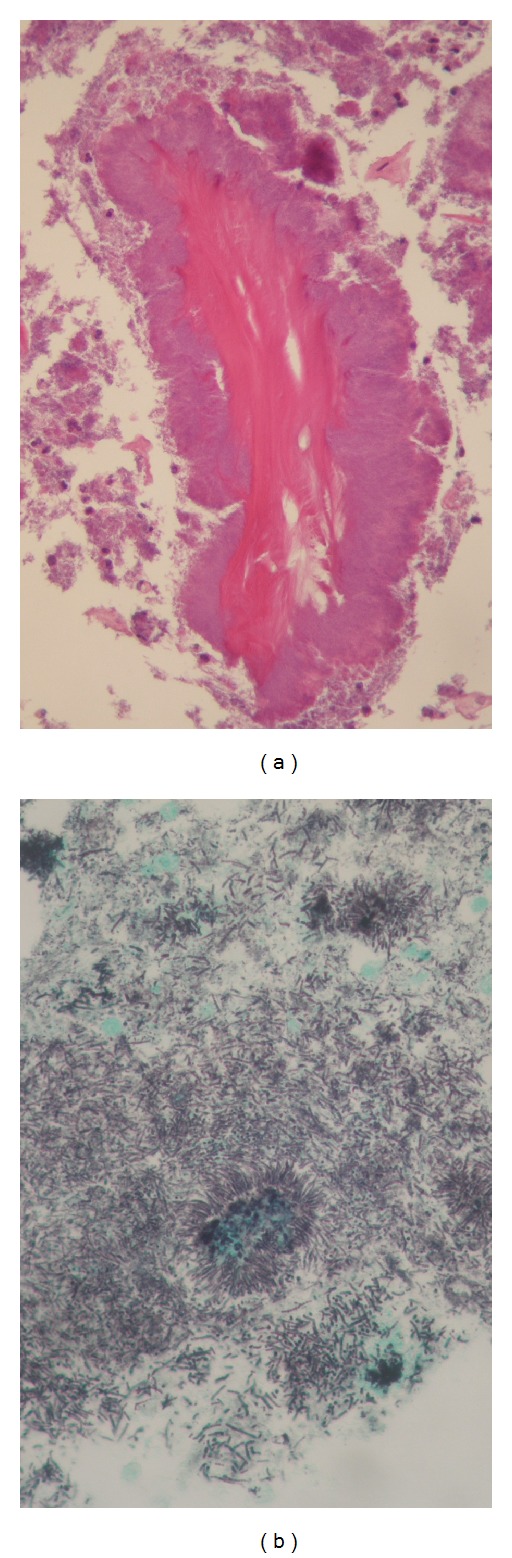
Actinomycotic druses (A, H.E. stain, 200x) and filaments (B, Grocott stain, 400x) detected from the sequestrum of the fracture site.

 When first examined, he had neither swelling in his cheek nor paresthesia in his lower lip. Postextraction hemorrhage of the mandibular first molar had already arrested. Instead, the clot was absent and the socket made the pale alveolar bone expose (Figure 1(a)). There was no redness or swelling in the regional gum and no mobility and percussion pain of the adjacent teeth. Panoramic radiograph showed neither abnormal consolidation nor ill-defined trabecular bone structure around the socket (Figure 1(b)). The clinical diagnosis was delayed healing of postextraction wound. White blood cell counts (WBC) were in normal range, and C-reactive protein (CRP) level slightly increased to 1.41 mg/dL. There was poor clinical evidence of acute inflammation (Figure 2). The information that the patient had been suffering from nonviral liver cirrhosis for 6 years and unremedied was not given at that time. Aspartate aminotransferase (AST) and alanine aminotransferase (ALT) were also in normal range. Clarithromycin (CAM) was administered for a week, but his spontaneous pain did not diminish. Mobility of the adjacent teeth and necrosis of the gum around the socket was present at 10 days after the first visit. We performed biopsy of the socket and extraction of the left mandibular second premolar, the results of which revealed no malignancy. CAM was administered for 10 more days. Computed tomography (CT) scans at 14 days after the first visit showed absorption of the cortical bone in the left mandibular molar region (Figure 3). Twenty-nine days after the first visit, the sequestrectomy and corticectomy of the left mandibular molar region and the extraction of the left mandibular first premolar and second molar were performed under general anesthesia. The surgical site was filled with gauze with pasta of dimethyl isopropyl azulene and clindamycin. Next day hyperbaric oxygen (HBO) utilization (2 atmosphere absolute, 90 minutes per day) begun for a total of 20 times. The patient was treated with intravenous penicillin for a week. After the sequestrectomy, spontaneous pain became bearable, and there was little clinical evidence of inflammation such as gum swelling or drainage. Fourty-two days after the surgery, he had swelling in his cheek. The patient was treated with intravenous piperacillin and clindamycin. Fourty-five days after the surgery, the mandible was fractured at the surgical site, and CT scans showed the bone resorption at the mandibular anterior teeth. Actinomycotic druses and filaments were detected from the sequestrum of the fracture site (Figure 4). Segmental resection and reconstruction were performed at 49 days after the first surgery. Actinomyces it was not detected any more from the resected mandible.

There has been no evidence of local recurrence 24 months after the treatment.

## 3. Discussion

Osteomyelitis of the jaws is caused in association with hematogenous germ spread, drug- or radiation-related, or local odontogenic or nonodontogenic processes [1]. Schafer states that dental infection is the most frequent cause of osteomyelitis of the jaws [4]. In the present case, panoramic radiograph at the time of preextraction of the left mandibular first molar showed neither abnormal consolidation nor ill-defined trabecular bone structure around the tooth, and the running of the inferior alveolar artery was clear. Osteomyelitis was esteemed to occur after the extraction, but the reason of the spontaneous pain which was the cause of the extraction was unclear.

 Panoramic radiograph at the initial visit to our hospital also showed no abnormal findings. Because of the clinical findings without mobility of the adjacent teeth, paresthesia in the left lower lip or swollen gums around the socket, the first diagnosis was just a delayed healing of the extraction wound as dry socket. However, the inflammation progressed rapidly, so we rediagnosed it as an acute osteomyelitis of the mandible. In the acute osteomyelitis, vascular compromise caused by the infective process occurs early in the course of the disease, making a cure unlikely unless medical management with the appropriate antibiotic is instituted within the first 3 days after the onset of the symptoms [1]. Early diagnosis is the key to prevent the disease from progressing. 

 Acute osteomyelitis of the jaw is often accompanied by symptoms as fever, malaise, facial cellulitis, trismus, and significant leukocytosis. In our case, although it had begun as an acute osteomyelitis, WBC was not remarkable and CRP level increased only slightly (Figure 2), and there was neither pus discharge nor swelling of the cheek until just before the fracture of the mandible. Rapidly progressing osteomyelitis which was highly resistant to treatments without typical symptoms like this case is extremely rare [5]. Osteomyelitis without typical symptoms made the final diagnosis delay and might bring the inflammation to progress. Systemic factors such as diabetes mellitus, agranulocytosis, leukemia, severe anemia, malnutrition, or alcohol abuse affect immune surveillance and lead to impairing the osteomyelitis [1]. The Cierny-Mader classification of long-bone osteomyelitis is based on the anatomy of bone infection and the physiology of the host [3]. Cierny described that not only the anatomic classification but also the condition of the host, regional vascularity, local milieu, and extent of necrosis would influence the natural history of the disease. In the present case, the patient had liver cirrhosis. Liver cirrhosis is one of the systemic factors in the classification that affect immune surveillance and metabolism. This patient's Child-Pugh score [6] was 8 points and the grade was B, significant functional compromise at the first surgery (Table 1). Child-Pugh grade can be used in patients with liver cirrhosis to assess the severity of the clinical condition [7]. Therefore, it was considered that impaired immunity introduced poor acute inflammatory reactions and the systemic compromise played a role in the asymptomatic and rapid progression of osteomyelitis.

 Identification of responsible microorganisms can be extremely difficult. Simply swabbing a suspected area is not appropriate. The process of obtaining suitable material for culture is fraught with potential danger of contamination from nearby oral site. In our case, actinomycotic druses and filaments were detected from the sequestrum of the fracture site, while they were not from the resected mandibular specimen. It was unclear whether their presence contributed to osteomyelitis development or they represented a secondary infection to the necrotic bone. However, it could not be denied to contribute to osteomyelitis development like in BRONJ [8–10]. Marx identified *Actinomyces *and other fastidious organisms such as *Eikenella* and *Arachnia* as pathogens in some of the more refractory forms of osteomyelitis of the jaws [11]. These organisms, in all likelihood, were contaminants with the original odontogenic microorganism invasion, but only became established after suboptimal therapeutics failed to eradicate all potential pathogens [2]. Robinson et al. [12] described that in the pediatric actinomycotic osteomyelitis the clinical manifestations are often subtle. Involvement by *Actinomyces *may be one of the causes that osteomyelitis progressed without typical symptoms in our case.

 In this paper, we report the case of the asymptomatic and rapid progression of osteomyelitis of the mandible. Depending on the predisposing factors, osteomyelitis progresses rapidly without typical symptoms. Correction of the underlying predisposing factors, early diagnosis and evaluating the therapeutic response of a multimodality treatment approach as needed would offer the best course of the disease.

## Figures and Tables

**Table 1:** Child-Pugh score in our case.

Measure	1 point	2 points	3 points	This case
Bilirubin (mg/dL)	<2	2-3	>3	2.1
Albumin (g/dL)	>3.5	2.8–3.5	<2.8	4
Prothrombin time (seconds)	1–3	4–6	>6	14.6
Ascites	None	Slight	Moderate	None
Encephalopathy (grade)	None	I-II	III-IV	None

Grade A = 5-6 points; well-compensated disease				8 points
Grade B = 7–9 points; significant functional compromise				
Grade C = 10–15 points; decompensated disease				
